# Hsp-90 and the biology of nematodes

**DOI:** 10.1186/1471-2148-9-254

**Published:** 2009-10-22

**Authors:** Nik AIIN Him, Victoria Gillan, Richard D Emes, Kirsty Maitland, Eileen Devaney

**Affiliations:** 1 Parasitology Group, Institute of Comparative Medicine, School of Veterinary Medicine, University of Glasgow, Bearsden Road, Glasgow G61 1QH, UK; 2 The School of Veterinary Medicine and Science, The University of Nottingham, Sutton Bonington Campus, Sutton Bonington, Leicestershire, LE12 5RD, UK

## Abstract

**Background:**

Hsp-90 from the free-living nematode *Caenorhabditis elegans *is unique in that it fails to bind to the specific Hsp-90 inhibitor, geldanamycin (GA). Here we surveyed 24 different free-living or parasitic nematodes with the aim of determining whether *C. elegans *Hsp-90 was the exception or the norm amongst the nematodes. We combined these data with codon evolution models in an attempt to identify whether *hsp-90 *from GA-binding and non-binding species has evolved under different evolutionary constraints.

**Results:**

We show that GA-binding is associated with life history: free-living nematodes and those parasitic species with free-living larval stages failed to bind GA. In contrast, obligate parasites and those worms in which the free-living stage in the environment is enclosed within a resistant egg, possess a GA-binding Hsp-90. We analysed Hsp-90 sequences from fifteen nematode species to determine whether nematode *hsp-90*s have undergone adaptive evolution that influences GA-binding. Our data provide evidence of rapid diversifying selection in the evolution of the *hsp-90 *gene along three separate lineages, and identified a number of residues showing significant evidence of adaptive evolution. However, we were unable to prove that the selection observed is correlated with the ability to bind geldanamycin or not.

**Conclusion:**

Hsp-90 is a multi-functional protein and the rapid evolution of the *hsp-90 *gene presumably correlates with other key cellular functions. Factors other than primary amino acid sequence may influence the ability of Hsp-90 to bind to geldanamycin.

## Background

Heat shock protein 90 (Hsp-90) is essential in all eukaryotes because of its role in chaperoning proteins such as kinases, growth factors and other signal transduction proteins [1]. As knock out of *hsp-90 *is generally lethal, specific inhibitors have been important for defining the cellular functions of Hsp-90. Of these, the best characterised is geldanamycin (GA) a naturally occurring ansamycin compound produced by *Streptomyces hygroscopicus *[2]. The mode of action of GA is well documented and the crystal structure of yeast Hsp-90 complexed to GA has been solved [3]. Most eukaryotic Hsp-90s are susceptible to inhibition with GA and will bind the drug in pull-down assays using GA linked to a solid support [4]. The exception to this rule is Hsp-90 (DAF-21) from the free-living nematode *Caenorhabditis elegans *(*Ce*), which fails to bind to GA in pull-down assays [5] and which shows no apparent ill-effect when maintained on a lawn of *S. hygroscopicus*. In contrast GA binds to Hsp-90 from the parasitic nematode *Brugia pahangi *(*Bp*) and is lethal to all life cycle stages [6]. The inability of *Ce*-Hsp-90 to bind GA was proposed to represent an example of adaptive evolution, as both *C. elegans *and the *Streptomyces *species that synthesizes GA are soil dwelling and so may share the same ecological niche [5]. In this study we adopted an integrated approach to investigating the possibility that nematode *hsp-90*s may have undergone adaptive evolution that influences GA-binding. We surveyed Hsp-90 from a range of free-living or parasitic nematodes for their ability to bind GA, and combined these data with statistical methods to compare substitution rates using maximum likelihood (ML) models of codon evolution [7,8], in an attempt to identify if significant evidence for adaptive diversifying evolution (*d*_N_/*d*_S _>1) relating to GA-binding exists. Specifically we investigated whether *hsp*-*90 *from GA-binding nematodes has evolved under differing evolutionary constraints compared to non-GA binding nematode species

Nematodes are an extensive group of animals that have evolved to colonise most ecological niches. Many nematodes are important parasites of humans, domestic animals and plants and are responsible for considerable economic losses worldwide [9]. Since the sequencing of the *C. elegans *genome, the genomes of several additional free-living nematodes have been sequenced, including two other species of *Caenorhabditis *(*briggsae *and *remanei*) and *Pristionchus pacificus *[10], a species that associates with beetles. Draft genome sequences are available for *C*. *brenneri *and *japonica*, and for the parasitic species *Trichinella spiralis *[11], *Brugia malayi *[12]*, Haemonchus contortus * and *Meloidogyne hapla *[13] and *incognita *[14], plant parasitic nematodes. Furthermore, sequencing projects are planned for several other important parasitic species . In addition EST projects have provided an abundance of sequence information on the phylum [15]. A phylogeny of the nematodes based on the small subunit ribosomal DNA sequences has been proposed [16] in which the nematodes are divided into five clades. Each of these clades contain parasitic species, suggesting that parasitism has arisen on multiple occasions during the evolution of the phylum. *C. elegans *is grouped in clade V, along with many important parasites of veterinary significance, while the filarial nematode *Brugia malayi *lies in clade III, as do some important ascarid parasites of humans and animals.

While free-living nematodes, such as Caenorhabditis species, often exhibit relatively simple life histories developing from egg to adult in one environment, parasitic species display a huge diversity in life cycles. Some parasitic species are transmitted direct from host to host and many of these have a free-living stage within the environment (e.g. the Trichostrongyles), while others, such as the filarial nematodes are obligate parasites. These worms have no free-living stages and are transmitted from host to host by the bite of an infected intermediate host, such as a mosquito. Likewise, the clade I species Trichinella spiralis is permanently parasitic and transmitted by ingestion of infected tissue. In this study, we set out to investigate whether the life history of a species was correlated with the ability of Hsp-90 to bind GA by analysing an additional twenty-four species of free-living or parasitic nematode, belonging to four of the five clades. These data were combined with an analysis of hsp-90 DNA sequences from a range of nematode species, in an attempt to determine whether the hsp-90 genes of GA-binding nematodes have significantly different ratios of non-synonomous (amino acid-changing) to synonomous (amino acid-retaining) nucleotide substitutions compared to non-binding species.

## Results

### GA pull-down assays

The ability of nematode Hsp-90 to bind to GA was assessed using a standard pull-down procedure in which GA bound to a solid support was used to select Hsp-90 from worm extract. The presence of Hsp-90 in the pull-down was assessed by immuno-blotting. Prior to use in pull-down experiments, extracts of each worm were probed with an anti-*B. pahangi *Hsp-90 antibody or with AC88, a monoclonal antibody to Hsp-90, to confirm that Hsp-90 could be detected on a blot. Extracts of three species failed to react with either antibody over a range of antibody concentrations and exposure times: *Trichinella spiralis*, *Trichuris muris *and *Anguillicolla crassus*. In addition, extracts of adult worms of *Haemonchus contortus *did not react with either antibody, despite the inclusion of *hsp-90 *sequences in the *Haemonchus *EST database with reasonable overlap to the 3' end of *Bp-*Hsp-90 that was used to make the antibody [6]. Extracts of adult *Haemonchus *were prepared in the presence of an additional protease inhibitor (E44) or directly by boiling in SDS-PAGE sample cocktail. Only when worms were extracted directly into sample cocktail could Hsp-90 be detected, suggesting that the aqueous extract may be subject to proteolytic degradation. In contrast, a soluble extract of the L3 stage reacted well with the *Brugia *antibody and was used in subsequent pull-down assays.

**Figure 1:**
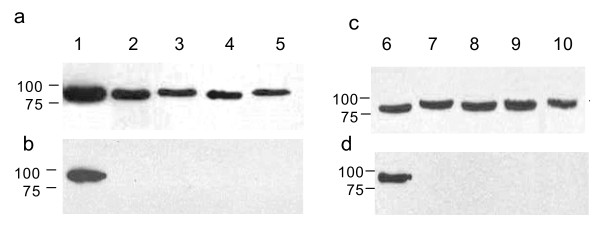
**Hsp-90 from clade V nematodes does not bind GA in a solid phase pull-down assay**. Extracts of various nematodes were analysed by immuno-blots (panels a and c) and by GA pull-down assay (panels b and d). Equivalent amounts of protein (determined by Bio-Rad protein assay) were analysed by SDS-PAGE on 10% gels and transferred to NCP. Blots were probed with 1:5000 rabbit anti-*Brugia *Hsp-90 antibody followed by 1:10,000 HRP-labelled anti-rabbit IgG. Bound antibody was detected by chemiluminesence. GA pull-down assays (panels b and d) were carried out with 500 μg of worm lysate using control or GA beads. Control beads were negative in all cases and are not shown. GA pull-down assays were analysed by SDS-PAGE and immuno-blotting as described above. Lane 1, *B. pahangi*, lane 2, *C. elegans *N2, lane 3, *C. elegans daf-21 *mutant, lane 4, *O. tipulae*, lane 5, *P. pacificus*, lane 6, *B. pahangi*, lane 7, *T. circumcincta*, lane 8, *N. brasiliensis*, lane 9, *H. polygyrus*, lane 10, *H. contortus*.

**Figure 2:**
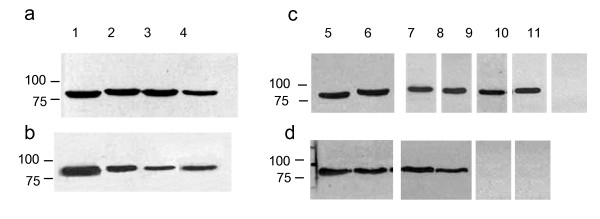
**Hsp-90 from some clade III nematodes binds to GA**. Extracts of various nematodes were analysed by immuno-blot (panels a and c) and by GA pull-down assay (panels b and d). Equivalent amounts of protein (determined by Bio-Rad protein assay) were analysed by SDS-PAGE on 10% gels and transferred to NCP. Blots were probed with 1:5000 rabbit anti-*Brugia *Hsp-90 antibody followed by 1:10,000 HRP-labelled anti-rabbit IgG. Bound antibody was detected by chemiluminesence. GA pull-down assays (panels b and d) were carried out with 500 μg of worm lysate using control or GA beads. Control beads were negative in all cases and are not shown. GA pull-down assays were analysed by SDS-PAGE and immuno-blotting as described above. Lane 1, *B. pahangi*, lane 2, *A. viteae*, lane 3 *D. immitis*, lane 4, *O. ochengi*, lane 5, *B. pahangi*, lane 6, *T. cati*, lane 7, *A. suum*, lane 8, *P. equorum*, lane 9, *A. simplex*, lane 10, *P. decipens*, lane 11, *A. crassus*.

Nine clade V nematodes were tested in the GA pull-down assay, five of which were free-living. These included *C. elegans *N2, *C. elegans *JT6130, a *daf-21 *mutant which carries a point mutation in *hsp-90 *(E292 to K), conferring a temperature-sensitive dauer-constitutive phenotype [17], *Caenorhabditis briggsae *(result not shown)*, Oscheia tipulae *and *Pristionchus pacificus*. In addition four parasitic species were tested: *Teladorsagia circumcincta*, *Nippostrongylus brasilensis*, *Heligmosomoides polygyrus *and *H. contortus*. Hsp-90 from all clade V species reacted positively with the anti-*Brugia *Hsp-90 antibody (Figure 1, panels a and c), but none bound to GA in the pull-down assay, whether from free-living or parasitic species (see Figure 1, panels b and d). Blots were exposed for extended periods of time but no positive reaction was observed, while the control *B. pahangi *lysate always yielded a strong positive result. A similar picture was observed with clade IV species, where Hsp-90 from *Strongyloides ratti*, two species of the plant parasite *Globodera *and the free-living *Panagrellus redivivus *all failed to bind GA (data not shown). For clade III nematodes, the situation was more diverse with Hsp-90 from all five filarial worms tested *B. pahangi*, *Acanthocheilonema viteae*, *Dirofilaria immitis*, *Onchocerca ochengi *and *Litomosoides sigmondontis *(result not shown) binding to GA (Figure 2, panel c). Hsp-90 from three ascarid species (*Toxocara cati*, *Ascaris suum *and *Parascaris equorum*) also bound to GA, but Hsp-90 from the related anisakids (*Anisakis simplex *and *Pseudoterranova decipens*) did not (Figure 2, panel d, lanes 9 & 10) while *A. crassus *failed to react with either antibody (Figure 2 panel c, lane 11). Where a positive signal was obtained in the pull-down assay, experiments were repeated in the presence of excess GA to block free binding sites. In all cases free GA blocked binding of Hsp-90 to the GA beads demonstrating the specificity of the interaction (data not shown).

Two species belonging to clade I were tested, *T. muris *and *T. spiralis *but neither reacted with the anti-Hsp-90 antibodies. For these species and for *A. crassus*, GA pull-down assays were analysed by Coomassie Blue staining. For *T. spiralis *only, the GA pull-down assay revealed a band of approximately 83 kDa that was not present using control beads. The binding to GA beads of the protein isolated from the *T. spiralis *extract was competed by pre-incubation of the extract with excess free GA. The band was excised from the gel, digested with trypsin and subjected to mass spectrometry at the Sir Henry Wellcome Functional Genomics Unit, University of Glasgow. MASCOT searches revealed multiple peptide hits to Hsp-90 from a variety of species with a high degree of significance (P < 0.05). Thus *T. spiralis *Hsp-90 binds to GA, while the GA-binding status of *A. crassus *and *T. muris *Hsp-90 remains uncertain. Table 1 summarises the data from the GA pull-down experiments.

**Table 1:** Summary of nematode species analysed and GA-binding properties

**Species**	**Clade**	**Life cycle stage**	**Life style**	**GA-binding**
*C. elegans*	V	Mixed stages	Free-living	-
*Ce daf-21 *mutant	V	Mixed stages	Free-living	-
*C. briggsae*	V	Mixed stages	Free-living	-
*P. pacificus*	V	Mixed stages	Free-living	-
*O. tipulae*	V	Mixed stages	Free-living	-
				
*H. contortus*	V	L3	Parasitic, FL larvae	-
*T. circumcincta*	V	Adult	Parasitic, FL larvae	-
*N. brasiliensis*	V	Adult	Parasitic, FL larvae	-
*H. polygyrus*	V	Adult	Parasitic, FL larvae	-
				
*S. ratti*	IV	Adult, FL	Parasitic or FL adult, FL larvae	-
				
*P. redivivus*	IV	Mixed stages	Free-living	-
				
*G. rostochiensis*	IV	J2	Plant parasitic	-
*G. pallida*	IV	J2	Plant parasitic	-
				
*B. pahangi*	III	Adult	Obligate parasite	+
*A. viteae*	III	Adult	Obligate parasite	+
*D. immitis*	III	Adult	Obligate parasite	+
*O. ochengi*	III	Adult, male only	Obligate parasite	+
				
*T. cati*	III	Adult	Parasitic, egg in environment	+
				
*P. equorum*	III	Adult	Parasitic, egg in environment	+
				
*A. suum*	III	Adult	Parasitic, egg in environment	+
				
*A. simplex*	III	L3	Marine, larvae in sea	-
*P. decipens*	III	L3	Marine, larvae in sea	-
*A. crassus*	III	Adult	Marine, larvae in sea	?
				
*T. spiralis*	I	L1	Obligate parasite	+
				
*T. muris*	I	Adult	Parasitic, egg in environment	?

FL, free-living; ? uncertain GA-binding status

### Bioinformatic analysis of nematode *hsp-90s*

Hsp-90 sequences from a variety of GA-binding and non-binding species of nematode were either retrieved from public databases or were cloned and sequenced as described in Methods. An alignment of the predicted amino acid sequences is given in supplementary data [see Additional file 1] and highlights the degree of conservation of Hsp-90, particularly over the N-terminal ATP-binding domain. Table 2 shows the pairwise similarity and identity between sequences used in the bioinformatic analyses described below. Full genomic and cDNA sequences were available for six species, *C. elegans*, *C. briggsae*, *P. pacificus*, *H. contortus*, *B. pahangi*, and *T. spiralis *allowing the comparison of *hsp-90 *gene structure between the different species. Exon-intron boundaries were mapped by comparison of genomic and cDNA sequences and are shown graphically [see Additional file 2]. Three introns are present in *C. elegans *and *C. briggsae *and the position of each is conserved. These are also perfectly conserved with those of *H. contortus *(equivalent to introns 1, 5 and 8 of *Hc-hsp-90*), although here the *Hc*-*hsp-90 *gene contains a total of eight introns. *B. pahangi *contains the largest numbers of introns (eleven), the position of three of which are conserved with *H. contortus*. A single intron (number 8 in *Hc-hsp-90*) is conserved in five of the six species analysed, the exception being *T. spiralis*.

**Table 2:** Pairwise percent similarities (bold) and identities (italic) between Hsp-90 peptide sequences of fifteen different nematodes calculated under the BLOSUM62 matrix image, generated using MatGAT [45].

	**1**	**2**	**3**	**4**	**5**	**6**	**7**	**8**	**9**	**10**	**11**	**12**	**13**	**14**	**15**
*1. B. pahangi*		*95.3*	*99.0*	*94.7*	*88.4*	*86.8*	*88.9*	*87.0*	*88.0*	*88.7*	*75.1*	*49.9*	*61.6*	*86.0*	*85.3*
*2. T. cati*	**98.7**		*95.7*	*97.6*	*88.6*	*87.5*	*88.8*	*86.9*	*87.6*	*88.5*	*74.3*	*49.0*	*60.8*	*85.9*	*85.3*
*3. L. sigmondontis*	**99.6**	**98.8**		*94.8*	*88.5*	*86.5*	*88.8*	*87.0*	*88.0*	*88.6*	*75.0*	*49.4*	*61.2*	*85.6*	*84.8*
*4. A. suum*	**97.6**	**98.8**	**97.8**		*88.2*	*86.6*	*88.1*	*86.6*	*87.2*	*88.1*	*73.9*	*48.7*	*60.2*	*85.4*	*84.6*
*5. H. contortus*	**94.5**	**94.4**	**94.5**	**94.0**		*90.0*	*90.7*	*90.3*	*89.6*	*89.9*	*76.6*	*49.0*	*60.3*	*83.7*	*82.7*
*6. P. pacificus*	**94.2**	**94.0**	**94.1**	**93.1**	**94.9**		*88.8*	*87.5*	*87.9*	*88.2*	*74.1*	*48.6*	*58.9*	*81.6*	*81.3*
*7. C. brenneri*	**95.1**	**94.8**	**95.0**	**94.0**	**95.8**	**95.1**		*95.8*	*97.9*	*98.7*	*81.5*	*49.6*	*59.6*	*82.6*	*81.8*
*8. C. elegans*	**93.9**	**93.7**	**93.8**	**92.9**	**95.6**	**94.2**	**98.5**		*97.0*	*96.0*	*79.3*	*49.8*	*59.9*	*83.8*	*80.8*
*9. C. remanei*	**94.2**	**94.1**	**94.2**	**93.2**	**95.8**	**94.8**	**99.1**	**99.1**		*97.8*	*80.4*	*49.8*	*59.9*	*82.8*	*80.8*
*10. C. briggsae*	**95.0**	**94.7**	**94.8**	**93.8**	**96.0**	**95.1**	**100.0**	**98.5**	**99.1**		*81.3*	*49.9*	*59.9*	*83.2*	*81.5*
*11. C. japonica*	**80.7**	**80.5**	**80.6**	**79.8**	**82.0**	**81.0**	**84.2**	**83.5**	**83.8**	**84.3**		*58.3*	*49.7*	*70.1*	*69.1*
*12. G. rostochiensis*	**55.1**	**54.7**	**54.9**	**54.0**	**55.0**	**54.3**	**54.9**	**54.7**	**55.2**	**54.9**	**64.7**		*66.3*	*54.3*	*51.9*
*13. G. pallida*	**68.3**	**68.1**	**68.2**	**67.3**	**68.2**	**67.3**	**68.3**	**68.0**	**68.7**	**68.3**	**65.6**	**70.2**		*67.1*	*64.1*
*14. H. glycines*	**94.0**	**94.6**	**94.0**	**93.7**	**92.1**	**91.5**	**92.5**	**91.8**	**92.2**	**92.5**	**78.5**	**56.2**	**69.6**		*88.6*
*15. M. Hapla*	**94.3**	**94.3**	**94.3**	**93.6**	**91.7**	**91.7**	**91.8**	**90.8**	**91.5**	**91.7**	**78.3**	**55.2**	**68.7**	**95.6**	

### Identifying sites and lineages subject to adaptive evolution

**Figure 3:**
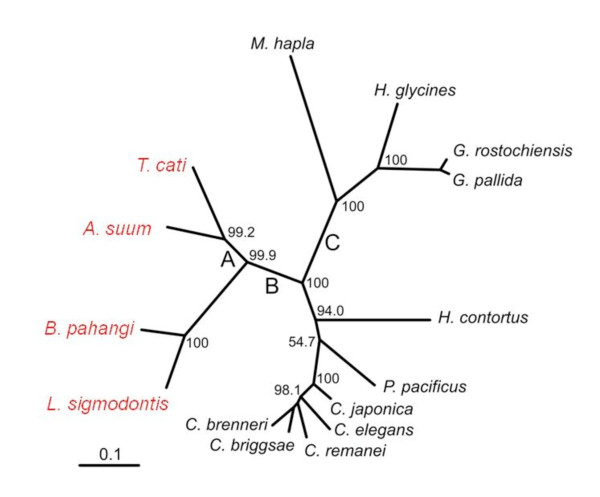
**Unrooted phylogenetic tree showing relationship of *hsp-90 *sequences from fifteen different nematode species**. The tree was generated using phyML [37] under the HKY substitution model. 1000 bootstrap replicates were performed and node labels represent percent bootstrap support. Branches A, B and C were shown to have significant evidence of positive selection. Scale bar shows substitutions per site.

For the gene-wide analysis, using either set of nested models of codon evolution (M1/M2 or M7/M8) did not identify any evidence of positive selection that was consistent across the phylogenetic tree (see Table 3). However, using the branch-sites methods which allow evolutionary rates to vary across the tree did show some interesting findings. Three branches showed significant evidence of adaptive evolution (dN/dS > 1) after Bonferroni correction (see Figure 3, labelled A, B, C). Along each of these branches, sites were detected with posterior probabilities > 0.95 of belonging to a dN/dS > 1 class (see Table 4). Along branch A (LRT of dN/dS > 1, p < 0.01) a single site 659S was identified (amino acid positions correspond to full length B. pahangi Hsp-90 AJ005785). On branch B (LRT of dN/dS > 1, p < 0.05), three sites with dN/dS > 1, 297S, 373S and 464S, were detected. On branch C, nine sites were detected 21S, 29S, 165V, 312S, 458S, 472Q, 485A, 525S and 584S. Importantly ten of these thirteen sites are serine residues which when examined are perfectly or largely conserved across all species in the alignment [see Additional file 1]. The identification of these ten sites as dN/dS > 1 results from the serine being encoded by either codons TCn or AGy. As the codon model of evolution assumes that mutations within a codon are independent, the change from TCn-AGy is parsimoniously interpreted as two separate non-synonymous substitutions (Z. Yang personal communication): e.g. TCC (Ser)->ACC (Thr)->AGC (Ser). The biological interpretation of these results suggests that the conservation of serine at these positions is functionally important and that this may be evidence for divergent evolution with rapid convergence back to the functionally stable serine residue. However, this cannot be proven by comparison of the extant species available to us and these sites are not considered further here. The remaining codons 165V, 472Q and 485A are inferred to be evolving rapidly along the lineage separating the plant parasitic nematodes from the animal parasitic and free-living species studied (branch C, Figure 3). Site 472Q is located in the middle region of the Hsp-90 protein and the codon is a conserved cysteine in the four plant parasitic nematodes and glutamine in all other species tested. Site 485A is also located in the middle region of the protein and is glutamine in the plant parasitic species and alanine in all other species. Only a single site, 165V, is predicted to be evolving under positive selection and is also located in the N terminal GA binding region of Hsp-90. Site 165V is a valine residue in the GA-binding species (L. sigmodontis, B. pahangi, A. sum and T. cati) but also in the non-GA binding P. pacificus and H. contortus. The residue is a phenylalanine in C. elegans and tyrosine in other Caenorhabditids analysed. To determine if these initial findings are consistent with the theory that GA-binding is associated with rapid diversifying evolution of the hsp-90 gene, we used a modified branch-site approach [18] to a priori separate the phylogenetic tree into two groups, GA-binders (B. pahangi, L. sigmodontis, A. suum and T. cati) vs non-GA-binders. Repeating the branch-sites analysis failed to provide significant evidence to suggest that an elevated dN/dS ratio associated with adaptive evolution was present in either of the two groups (see Table 5).

**Table 3:** Statistical analysis of sites subject to adaptive evolution using nested models of codon evolution (M1/M2 or M7/M8)

		**LRT statistic**		**Parameter estimates**			**Sites under positive selection**	
				**% Sites with d_N_/d_S_> 1**				

Gene	N	M1 vs. M2	M7 vs. M8	M2	M8	d_N_/d_S_	M2	M8
Hsp90	15	0.00	0.00	n/a	n/a	1.00	None	None

n/a not available

**Table 4:** Statistical analysis of branches showing significant evidence of adaptive evolution

			**Significance of tests for positive selection**		
**Gene**	**Number of sequences**	**Number of codons**	**Sites**		**Branch sites All branches**
			**M2**	**M8**	

Hsp90	15	687	n/s	n/s	2 branches (p < 0.05, B & C Figure 3) 1 branch (p < 0.01, A Figure 3

n/s not significant

**Table 5:** Branch-site analysis of *hsp-90 *sequences grouped according to GA-binding status

	**Number of branches**			**Parameter estimates under free model**			**d_N_/d_S_**		
**Clade**	**All**	**foreground**	**LRT statistic**	**p0^a^**	**p1^b^**	**p2^c^**	**background**	**foreground**	**Sites under positive selection**

GA binders	27	7	0.00	0.984	0.012	0.004	0.014	1.00	n/a
GA non-binders	27	20	0.00	0.978	0.004	0.18	0.014	1.00	n/a

n/a not available. Number of branches: count of branches partitioned as foreground. LRT statistic: 2 times log likelihood difference of fixed versus free nested models.

^a ^Proportion of sites in category p0 where selection is purifying throughout the tree (0 < ω_0 _< 1).

^b ^Proportion of sites in category p1 where selection is neutral throughout the tree (ω_1 _= 1).

^c ^Proportion of sites in category p2 where selection is positive in foreground branches; sum of category 2a: purifying selection in background but positive selection in foreground (0 < ω_0 _< 1, ω_2 _> 1); and category 2b: neutral selection in background but positive selection in foreground (ω_1 _= 1, ω_2 _> 1

## Discussion

The results of our analysis of Hsp-90 from a range of parasitic and free-living nematodes has revealed an interesting heterogeneity in the ability to bind GA. None of the free-living species tested, nor any of the parasitic species which have free-living larval stages in the environment bound to GA. These included animal parasites belonging to clade IV, such as *Strongyloides*, and those such as *Haemonchus *and *Nippostrongylus *belonging to clade V. These parasites are generally gut-dwelling and are transmitted between hosts when eggs, passed out in the faeces of the infected animal, hatch to release free-living larval stages. Following a period of development in the environment, the L3 stage infects a new host. The plant parasites tested, *Globodera *spp., also failed to bind GA. These are commonly known as potato cyst nematodes, and live in the soil where the infective second-stage juvenile worms (J2) attack the roots of susceptible plants. A cyst containing eggs is formed from the body of the dead female worm and under appropriate conditions these will hatch to release the J2 stage worms. These penetrate the roots, start to feed and eventually develop into adults.

The clade III nematodes analysed produced an interesting dichotomy in results, containing both GA-binding and non-binding species. Hsp-90 from the five filarial parasites assayed bound to GA; filarial worms are obligate parasites, transmitted between hosts by the bite of an infected intermediate host, such as mosquitoes or other arthropods. In keeping with this result, Hsp-90 from another obligate parasite *T. spiralis *also bound GA. *T. spiralis *is a clade I species commonly transmitted via ingestion of infected muscle containing the encysted L1 stages. Thus neither filarial worms nor *T. spiralis *have free-living stages in the environment.

The clade III Ascarid parasites also provided an intriguing comparison, as those worms which bound GA (*Toxocara*, *Ascaris *and *Parascaris*) all have free-living stages outside the host, but are enclosed within a thick impermeable egg-shell while in the environment. Transmission to the new host is by ingestion of the egg containing the infective larva or via a paratenic host (in some species such as *Toxocara*). Interestingly, in the original report on the isolation of GA [2], the in vivo efficacy of GA against the mouse pinworm *Syphacia obvelata *is recorded, suggesting that this parasite may also express a GA-binding Hsp-90. In keeping with this observation, pinworms belong to clade III (Oxyuroids) and are transmitted by ingestion of a resistant egg containing the infective larval stage.

Two of the three clade III species associated with the marine environment (*Anisakis *and *Pseudoterranova*) failed to bind GA. Both parasites have complex life cycles involving one or more intermediate and/or paratenic hosts. The free-living larval stages are ingested by marine crustaceans, which are then eaten by fish, which are subsequently ingested by marine mammals such as seals (*Pseudoterranova*) or cetaceans (*Anisakis*). *A. crassus *belongs to the superfamily Dracunuloidea and parasitises the swim bladder of eels, but also has a free-swimming larval stage. As extracts of *A. crassus *(and *T. muris*) failed to react with either antibody, the GA-binding status of these species remains unresolved. An additional proviso of our data is that in some cases, larval stages were analysed and it is possible that larval and adult worms may display different GA-binding characteristics.

Our data suggest that differences in the ability of nematode Hsp-90 to bind to GA are correlated with particular life histories. Only obligate parasites or those which have free-living stages in the environment enclosed within a protective egg-shell possess a GA-binding Hsp-90, while Hsp-90 from free-living nematodes or from those parasitic species that have free-living larval stages in the environment do not bind GA. The adaptive evolution hypothesis would argue that organisms sharing the same niche as the GA-producing *Streptomyces *should fail to bind GA and the results from the pull-down experiments broadly support this hypothesis. In order to further explore this theory, we analysed *hsp-90 *sequences from fifteen different species of nematode in an attempt to identify codons that have undergone adaptive Darwinian evolution within the GA-resistant species. The results of these analyses provided strong evidence for rapid adaptive evolution along three separate lineages and identified a number of residues showing significant evidence of adaptive evolution. However, the model suggests that ten of the thirteen codons identified as sites of adaptive evolution had undergone at least two separate non-synonomous substitutions resulting in the conservation of a serine residue, implying that these sites may be critical for Hsp90 function. In mammalian Hsp-90α and β, both serine and threonine residues are known to be targets of phosphorylation [19,20], a modification that is essential for some functions of Hsp-90 [21,22]. Of the remaining three residues (165V, 472Q and 485A) showing significant evidence of adaptive evolution (posterior probability > 0.95 of *d*_N_/*d*_S _> 1), only a single site, 165V, was located in the amino terminal section of the Hsp-90 protein, known to be the position of GA binding. However, variation at this site does not differentiate between GA-binding and non-GA binding species. The residue is a valine in the GA binding species, but also in the non-GA binding species *P. pacificus *and *H. contortus*. Comparison of the amino acid sequences of Hsp-90 from GA-binding species and non-binding species identified only four amino acid positions that are conserved within either the GA-binding or the non-binding species, but that differ between the groups. These are: K259 (K GA-binders, T non-binders), F447 (F GA-binders, Y non-binders), F475 (F GA-binders, Y non-binders), V624 (V GA-binders, I non-binders) (all amino acid positions correspond to full length *B. pahangi *Hsp-90 [Genbank:AJ005785]). However, none of these substitutions reside in the N terminal region. Comparison of each of these positions in *Brugia *and *Saccharomyces cerevisiae*, which binds GA [23], shows that the equivalent amino acids in yeast are conserved with the non-GA-binding nematode species. Thus whilst evidence for rapid diversifying selection exists in the evolution of the *hsp-90 *gene (as evidenced by the significance of the branch-site tests) and most likely occurred early in nematode divergence, we have been unable to prove with confidence that the selection seen correlates with a change in the GA-binding function of Hsp-90. As Hsp-90 is a multi-functional protein in eukaryotic cells, the observed rapid evolution of the *hsp-90 *gene likely correlates with some other key function, rather than the ability to bind GA.

In *S. cerevisiae*, point mutations in *hsp-90 *alter the sensitivity to GA and to Radicicol, an alternative inhibitor of Hsp-90 function [23]; e.g. A587T and T101I are hypersensitive to GA, with T22I, G313S and E381K showing a lesser effect. However, additional factors are also known to influence sensitivity to GA in yeast, including the presence of co-chaperones such as Sti1p (mammalian Hop) and Sba1p (mammalian p23). Yeast deficient in Sti1p or Sba1p show increased sensitivity to GA [23,24]. *C. elegans *expresses homologues of both co-chaperones [25] and a recent study identified STI-1 as an Hsp-90 interacting protein in *C. elegans *[26]. However, whether *sti-1 *null animals have an altered response to GA has yet to be explored.

While analysis of *daf-21 *mutants and RNAi studies in *C. elegans *clearly show that Hsp-90 is essential in the worm, *C. elegans *is not susceptible to GA and, indeed, in the micro-environment of a Petri dish, can grow perfectly well when fed on a culture of *S. hygroscopicus *[5]. However, it remains unclear whether *C. elegans *would interact with the GA-producing *S. hygroscopicus *in the environment. Although both organisms may be present in the soil, whether they share the same spatial and temporal niches is unknown. Indeed, the ecology of *C. elegans *in the wild is not well understood, despite its extensive use in the laboratory [27]. Although *C. elegans *can be found in soil, it appears that the predominant stage observed in the wild is the dauer larvae, typically found in organic-rich environments such as compost, or in association with invertebrates such as snails and isopods [28]. Dauer stages are resistant and non-feeding and the natural food source of *C. elegans *in the wild is unknown. *C. elegans *possesses a sensitive chemosensory system that, at least in the laboratory setting, allows the worm to differentiate between bacterial food sources and potential pathogens [29]. Thus in the environment, worms could potentially avoid bacteria producing noxious compounds.

Many Hsp90 inhibitors are natural compounds produced by micro-organisms and recent studies have provided an intriguing insight into the mechanisms by which the organisms that produce Hsp90 inhibitors can themselves survive. For example, in the yeast *Humicola fuscoatra*, which produces Radicicol, a single conserved amino acid substitution (L33I) in Hsp-90, is sufficient to alter the binding of Radicicol, without influencing the ability to bind ATP or GA [30]. Here, a leucine to isoleucine substitution alters the size of the hydrophobic drug-binding pocket increasing the binding of H_2_O molecules and hydration of the drug. Such findings serve to emphasise that very subtle alterations in amino acid residues can have major implications on drug binding efficacy and highlight the importance of understanding the structural basis of drug binding, which is not always apparent from analysis of DNA sequences.

## Conclusion

Our results demonstrate that *C. elegans *Hsp-90 is not unique in its low affinity for GA; despite a relatively high level of sequence conservation, nematode Hsp-90s show functional diversity in relation to GA-binding. The ability to bind to GA is associated with the life history of a species; free-living nematodes and those parasitic species which have a free-living larval stage in the environment do not bind GA, while obligate parasites or those parasitic species which have free-living stages enclosed within a resistant egg, do bind GA. As *S. hygroscopicus*, the micro-organism that synthesizes GA, is found in soil, and as Hsp-90 is an essential molecule in all eukaryotes, these observations could be consistent with adaptive evolution of *hsp-90 *sequences. Analysis of *hsp-90 *sequences from fifteen different nematode species provided evidence of rapid diversifying selection along three separate branches. However, the selection observed did not correlate with the ability to bind GA or not.

## Methods

### Worm material

The following species of free-living nematode were used *C. elegans *N2 strain, *C. elegans *strain JT6130, which carries a point mutation in *daf-21 *(*hsp-90*), *Caenorhabditis briggsae, Oscheia tipulae*, *Pristionchus pacificus *(all clade V, obtained from the *C. elegans *Genetics Center) and *Panagrellus redivivus *(clade IV). Parasitic species analysed included *Trichinella spiralis *and *Trichuris muris *(both clade I), and the following clade III species: *Brugia pahangi*, *Dirofilaria immitis, Onchocerca ochengi*, *Acanthocheilonema viteae*, *Litomosoides sigmondontis*, *Toxocara cati, Ascaris suum, Parascaris equorum*, *Anisakis simplex, Pseudoterranova decipens *and *Anguillicola crassus*. Clade IV species included *Strongyloides ratti*, *Globodera rostochiensis *and *G. pallida*. Clade V species included *Nippostrongylus brasilensis*, *Heligmosomoides polygyrus*, *Teleodorsagia circumcincta *and *Haemonchus contortus*. Mixed life cycle stages were used for all the free-living nematodes while for parasitic species, usage was determined by the availability of particular life cycle stages; in most cases adult males and females were analysed with the following exceptions: *Globodera *sp. J2 stages; *H. contortus*, L3; *T. spiralis *L1 muscle stage larvae; *A. simplex *and *P. decipens *L3. *S. ratti *adults were free-living rather than parasitic females [31] and for *O. ochengi *only male worms were used.

### Maintenance of nematodes

*C. elegans *N2 strain, *C. briggsae, O. tipulae *and *P. pacificus *were maintained at 20°C on NGM plates with OP50 as a bacterial food source [32]. Worms were washed off plates with M9 buffer (3 g KH_2_PO_4_, 6 g Na_2_HPO_4_, 5 g NaCl, 1 ml 1 M MgSO_4 _in 1 litre ddH_2_O) and collected by centrifugation. *P. redivivus *worms were maintained at room temperature on plates containing an oat-based medium. The cooked oats were poured into plates and the worms placed on the surface. As their population blooms, the worms move onto the lid, from where they can be collected by rinsing the lids with M9 buffer. Worms were then washed several times in M9 buffer by centrifugation.

Adult stages of all parasitic species were obtained from their respective hosts and frozen as soon as possible at -80°C. As no signal on a western blot could be obtained using anti- Hsp-90 antibodies to probe extracts of adult *H. contortus*, L3 of this species were obtained by harvesting eggs from infected sheep faeces and allowing these to develop [33]. For *Globodera *species, cysts were rehydrated in tap water for several days and then hatched in tomato root exudate (kindly provided by J. Jones, Scottish Plant Breeding Institute, Invergowrie). Cysts were checked daily for hatched J2 stages. These were removed by pipetting, washed in ddH_2_0 and purified from debris by flotation on 60% sucrose. For *T. spiralis*, muscle-stage larvae (L1) were obtained by digestion of infected mouse muscle using standard methods [34]. Larval stages (L3) of *A. simplex *and *P. decipens *were obtained from infected fish.

### Preparation of worm lysates

Lysates of worms were prepared in ice-cold TNES buffer (50 mM Tris-HCl, pH 7.4, 100 mM NaCl, 2 mM EDTA, 1% Igepal CA-630). Protease inhibitors were added prior to use (Complete Mini Protease Inhibitor Cocktail, Roche Diagnostics). Worms were ground to a fine powder using a pestle and mortar containing liquid Nitrogen. Extracts were resuspended in 1.0 ml TNES, the mortar was washed out with 0.5 ml TNES, and the extract was pelleted at 14000 g for 15 minutes at 4°C. The supernatant was retained and assayed for protein concentration at OD 595 nm using the Bio-Rad Protein assay. Lysates of D. immitis and O. ochengi were prepared using a small glass homogeniser containing 100 μl of ice-cold TNES and the supernatant processed as described above.

### Preparation of GA solid support and binding assay

1 mg GA (InvivoGen) was derivatised using 1,6-hexanediamine to yield 17-hexamethylenediamine-17 demethoxygeldanamycin [4]. After aqueous extraction, the mixture was dried under nitrogen then re-dissolved in 400 μl of DMSO. The product was incubated with 400 μl of washed Affi-Beads (Bio-Rad) for 4 h in the dark at room temperature. Beads were washed in TNES and then were rocked overnight at 4°C in 1.0 ml of ethanolamine (1 M, pH 8.3) to block residual binding sites. Prior to use, beads were blocked in 1% BSA in TNES for 1 h at room temperature. Control beads were incubated with DMSO alone.

300-500 μg of total protein in a volume of 300 μl was mixed with 50 μl packed volume of GA or control beads for 2.5 hours at 4°C. The beads were washed extensively in TNES by centrifugation and bound proteins were eluted by boiling in SDS-PAGE sample cocktail (0.125 M Tris, pH 6.8, 0.2 M dithiothreitol, 4% SDS, 20% glycerol, 0.042% bromophenol blue). Aliquots of the input fraction and protein eluted from control and GA beads were run on 10% SDS-PAGE gels and analysed by western blotting. Blots were probed with a 1:5000 dilution of an antibody raised to a C-terminal fragment of recombinant *B. pahangi *Hsp-90, as detailed previously [6], followed by 1:10000 dilution of HRP-labelled anti-rabbit IgG (Sigma). Bound antibody was detected by chemiluminescence using the Super Signal ^® ^West Pico Kit (Thermo Scientific). If no signal or only a weak signal was observed, blots were repeated using the AC88 Hsp-90 monoclonal antibody (a kind gift of D. Toft) as the primary antibody at 1:500 dilution followed by anti-mouse IgG (Sigma) at 1:10000. AC88 recognises an epitope in the C-terminus of Hsp-90 and reacts with Hsp-90 from many different organisms. In all experiments, a lysate of *B. pahangi *was used as a positive control. The specificity of GA binding was determined in subsequent experiments by pre-incubating worm extracts with 20 μM GA prior to incubation with the GA beads.

### Sequence and bioinformatic analysis

*hsp-90 *sequences from *B. pahangi *[GenBank:AJ005785], *H. glycines *[GenBank:AF461150], *M. hapla *[GenBank:AAT35588], *C. elegans *[GenBank:C47E8.5] and *C. briggsae *[GenBank:CBG040560] were obtained from the NCBI. *C. brenneri*, *C. japonica *and *C. remanei hsp-90 *sequences were obtained by carrying out a tBLASTn search at  using the *Ce-*DAF-21 amino acid sequence to retrieve full length cDNA sequences for each *Caenorhabditis *species.

The full-length sequence of *T. spiralis hsp-90 *was identified by a tBLASTn search of the available genome sequence at  with the *Ce-*DAF-21 amino acid sequence. A significant match was obtained to contig 9.2, the sequence of which was obtained from Amy Yu at Washington University School of Medicine. For *P. pacificus*, the full-length *hsp-90 *sequence was obtained by tBLASTn search of the sequence available at  using the *Ce-*DAF-21 amino acid sequence, resulting in the retrieval of contig 4.9. For *Trichinella *and *Pristionchus*, intron and exon sites were identified using Genewise in EXPASY, allowing assembly of the full-length predicted cDNA sequence. For *H. contortus*, the *hsp-90 *sequence was assembled by carrying out a tBLASTn search at  using the *Ce-*DAF-21 amino acid sequence to retrieve contig _0076156, as detailed previously [35]. The sequence was submitted to GenBank [GenBank:FJ717747].

For *A. suum *a partial *hsp-90 *sequence was obtained by carrying out a tBLASTn search at  using the *Ce-*DAF-21 amino acid sequence and retrieving EST sequence kj94a07.y1, which contained 768 bp of 5' *A. suum Hsp-90 *sequence. A primer was designed to the 5' end of the gene, 5'-ATGTCTGAGCAACAGCCTGAAGGA-3' and used in combination with a series of degenerate primers, designed over the conserved MEEVD region, to amplify the full length *hsp-90 *coding sequence from adult *A. suum *cDNA. One 3' degenerate primer was more successful than others and was used in all subsequent reactions, 5'-ANTCNACNTCNTCCATNCGNGANGC-3'. Full length *A. suum hsp-90 *was cloned into pCR2.1 TOPO (Invitrogen) and sequenced by automated DNA sequencing using vector and gene specific primers (MWG Biotech) and assigned an accession number [GenBank:FJ824589].

For *L. sigmodontis *and *T. canis *partial *hsp-90 *sequences were obtained by carrying out a tBLASTn search at  using the *Bp-*HSP-90 amino acid sequence. A 721 bp EST was retrieved which contained 676 bp of 3' *L. sigmodontis hsp-90 *sequence. As the 5' sequence was missing, an SL1 primer was used (5'-GGTTTAATTACCCAAGTTTGA-3') along with a primer designed to the 3' end of the *L. sigmodontis *sequence, 5'-TTAATCAACTTCTTCCATCCTCG-3'. The full length *L. sigmodontis hsp-90 *was amplified from adult *L. sigmondontis *cDNA, cloned into pCR2.1 TOPO (Invitrogen), sequenced as above and assigned an accession number [GenBank:FJ824588].

For *T. canis*, an EST of 647 bp was identified containing the 5' region of the gene. As the only biological material available was from the sister species *T. cati*, PCR was carried out using a primer designed to the 5' end of the *T. canis hsp-90 *sequence (5'-ATGTCCGAGTTTCAACAACAACCAG-3') together with the degenerate 3' primer described previously (see above) on cDNA extracted from adult *T. cati *worms. The full-length *T. cati hsp-90 *was cloned into pCR2.1 TOPO (Invitrogen), sequenced as above and assigned an accession number [GenBank:FJ824590]. All sequences were compiled from multiple reads in both directions and line-ups and annotations were carried out using Contig express and Vector NTI.

Partial *hsp-90 *sequences of *X. index *(1325 bp), *G. pallida *(685 bp) and *G. rostochiensis *(1394 bp) were obtained by carrying out a tBLASTn search at  or  using the *Bp-*HSP-90 amino acid sequence. For those species for which both full-length cDNA and genomic *hsp-90 *sequences were available, intron/exon boundaries were identified in genomic DNA sequences using Genewise in EXPASY and their positions mapped onto cDNA sequences.

### Identifying sites and lineages subject to adaptive evolution

Peptide sequences were generated from translated nucleotide sequences of *hsp-90 *from seventeen species of nematode *C. remanei, C. briggsae, C. brenneri, C. elegans, C. japonica, P. pacificus, H. contortus, L. sigmodontis, B. pahangi, A. suum, T. cati, M. hapla, Heterodera glycines, G. pallida, G. rostochiensis, T. spiralis *and *Xiphinema index*. These were aligned using Prank [36] and the aligned peptide sequence was back translated to generate an aligned nucleotide sequence. A phylogenetic tree, generated with phyML [37] under the HKY substitution model [38], was used as the starting phylogeny for the codeML program of the Phylogenetic Analysis by Maximum Likelihood (PAML) suite [39]. A phylogenetic tree generated under the M0 model inferred that branches leading to two of the species, *T. spiralis *and *X. index*, exhibited much larger divergence (> 3 substitutions per codon). To avoid potential problems of saturation at high divergence these species were removed from further analyses. Sequences from the remaining fifteen species were realigned with Prank and a maximum likelihood phylogenetic tree was again generated using phyML. To determine if evidence for adaptive evolution (*d*_N_/*d*_S _>1) exists in these sequences, three tests of selection were conducted to compare the ratio of non-synonymous to synonymous substitutions (*d*_N_/*d*_S_). (1) Site analysis using a likelihood ratio test (LRT) to compare the codeML nested models M1/M2 and M7/M8 which assume a uniform evolutionary rate across all branches of the tree [40,41]. (2) Branch-sites methods, which allow for differential rates of evolution across different lineages [7,8]. Using the branch-sites methods each individual branch was analysed. (3) Species were also grouped into GA-binders or non-binders and compared using the branch-sites method, as such comparisons can improve the power of analysis if the underlying biological assumption to group the branches is sound [18]. In all cases multiple independent runs of the CodeML program were conducted (minimum triplicate) to ensure convergence of maximum likelihood estimates of parameters. Where multiple tests were conducted, the type I error rate was controlled by Bonferroni correction [42] (See Table 3). Hsp-90 amino acid sequences from nematode species used in the bioinformatic analysis were also aligned using ClustalW [43] and coloured using the default residue groupings of CHROMA [44].

## Abbreviations

Hsp: heat shock protein; GA: geldanamycin; SDS: PAGE- sodium dodecyl sulphate-polyacrylamide gel electrophoresis; Ce: *C. elegans*; Bp: *B. pahangi*

## Authors' contributions

NH carried out the pull-down assays; VG identified and sequenced *hsp90 *genes; KM helped with the pull-down assays; RDE undertook the *d*_N_/*d*_S _analysis; ED conceived and coordinated the study. NH, VG, RDE and ED contributed to the manuscript. All authors provided comments on the manuscript and read and approved the final draft.

## Supplementary Material

Additional file 1 **Comparison of Hsp-90 sequences from fifteen nematode species used for phylogenetic analysis**. Consensus coloured alignment of nematode Hsp-90 amino acid sequences using default residue groupings of CHROMA. Sequence positions are numbered according to available protein data; see Methods section of manuscript for details of accession numbers. Positions of protein domains were determined by alignment of *S. cerevisiae *Hsp90. Click here for file

Additional file 2 **Genomic structure of nematode *hsp-90 *genes**. The figure shows intron positions in genomic DNA sequences of *hsp-90 *from *B. pahangi*, *H. contortus*, *C. elegans*, *C. briggsae*, *P. pacificus *and *T. spiralis*. Introns were mapped by comparison of cDNA and genomic DNA sequences. Intron positions were marked by hand and introns with common positions are shaded. Numbers refer to the length of cDNA sequences of each gene. Click here for file
