# Supplementary material for: Hsp-90 and the biology of nematodes
Source: BMC Evol Biol. 2009 Oct 22;9:254. doi: 10.1186/1471-2148-9-254 (PMC2771018; doi:10.1186/1471-2148-9-254)
Supplement: Additional file 1 — Comparison of Hsp-90 sequences from fifteen nematode species used for phylogenetic analysis. Consensus coloured alignment of nematode Hsp-90 amino acid sequences using default residue groupings of CHROMA. Sequence positions are numbered according to available protein data; see Methods section of manuscript for details of accession numbers. Positions of protein domains were determined by alignment of S. cerevisiae Hsp90. [file 1471-2148-9-254-S1.PDF]

```
B.pahangi          (10)  FAFQAEIAQLMSLIINTFYSNKEIFLRELISNSSDALDKIRYQALTEPAELETGKELYIKITPNKADKTLTIMDTGIGMT
T.cati             (13)  FAFQAEIAQLMSLIINTFYSNKEIFLRELISNSSDALDKIRYQALTEPSELDTGKELYIKITPNKADKTLTILDTGIGMT
L.sigmondontis     (10)  FAFQAEIAQLMSLIINTFYSNKEIFLRELISNSSDALDKIRYQALTEPAELETGKELYIKITPNKADKTLTIMDTGIGMT
A.suum             (11)  FAFQAEIAQLMSLIINTFYSNKEIFLRELISNSSDALDKIRYQALTEPSELDTGKELFIKITPIKADKTLTILDTGIGMT
H.contortus         (9)  FAFQAEIAQLMSLIINTFYSNKGIFLRELISNSSDALDKIRYQALTEPGELDTGKELYIKIIPNKEDKTLTIIDTGIGMT
P.pacificus         (3)  FAFQAEIAQLMSLIINTFYSNKEIFLRELVSNASDALDKIRYQALTEPSELDSGKELYIKITPNKAEKTLTIFDSGIGMT
C.brenneri          (8)  FAFQAEIAQLMSLIINTFYSNKEIYLRELISNASDALDKIRYQALTEPSELDTGKELYIKITPNKEEKTLTIMDTGIGMT
C.elegans           (8)  FAFQAEIAQLMSLIINTFYSNKEIYLRELISNASDALDKIRYQALTEPSELDTGKELFIKITPNKEEKTLTIMDTGIGMT
C.remanei           (8)  FAFQAEIAQLMSLIINTFYSNKEIYLRELISNASDALDKIRYQALTEPSELDTGKELFIKITPNKEEKTLTIMDTGIGMT
C.briggsae          (8)  FAFQAEIAQLMSLIINTFYSNKEIYLRELISNASDALDKIRYQALTEPSELDTGKELFIKITPNKEEKTLTIMDTGIGMT
C.japonica               --------------------------------------------------------------------------------
G.rostochiensis          --------------------------------------------------------------------------------
G.pallida          (24)  ---------------NTFYSNKEIFLRELISNSSDALDKIRYQALTDATQMETGKELYIKIVPNKADKTLTIIDTGIGMT
H.glycines          (8)  FAFQAEIAQLMSLIINTFYSNKEIFLRELISNSSDALDKIRYQALTDPSQMESGKELFIKIVPNKADKTLTIIDTGIGMT
M.hapla             (3)  FAFQAEIAQLMSLIINTFYSNKEIFLRELISNSSDALDKIRYQALTDPAQLESGKDLYIKVIPNKADKTLTIVDTGIGMT
Consensus/80%            FAFQAEIAQLMSLIINTFYSNKEIaLRELISNtSDALDKIRYQALT-Ptpb-*GKELaIKIhPNK.-KTLTIbDTGIGMT

B.pahangi          (90)  KADLVNNLGTIAKSGTKAFMEALQAGADISMIGQFGAGFYSAFLVADKVVVASKHNDDDCYQWESSAGGSFIIRQVNDPE
T.cati             (93)  KADLVNNLGTIAKSGTKAFMEALQAGADISMIGQFGVGFYSAFLVADRVIVTSKHNDDDCYQWESSAGGSFIIRQVNDPE
L.sigmondontis     (90)  KADLVNNLGTIAKSGTKAFMEALQAGADISMIGQFGVGFYSAFLVADKVVVASKHNDDDCYQWESSAGGSFIIRQVNDPE
A.suum             (91)  KADLVNNLGTIAKSGTKAFMEALQAGADISMIGQFGVGFYSAFLVADRVVVTSKHNDDDCYQWESSAGGSFIIRQVNDPE
H.contortus        (89)  KADLVNNLGTIAKSGTKAFMEALQAGADISMIGQFGVGFYSAFLVADRVVVTSKHNDDDCYQWESSAGGSFVV-AVNDPE
P.pacificus        (83)  KADLVNNLGTIAKSGTKAFMEALQAGADISMIGQFGVGFYSAFLVADKVVVTSKHNDDDCHVWESSAGGSFTV-TVNDPE
C.brenneri         (88)  KADLVNNLGTIAKSGTKAFMEALQAGADISMIGQFGVGFYSAFLVADKVVVTSKNNDDESYQWESSAGGSFVVRPYNDPE
C.elegans          (88)  KADLVNNLGTIAKSGTKAFMEALQAGADISMIGQFGVGFYSAFLVADKVVVTSKNNDDDSYQWESSAGGSFVVRPFNDPE
C.remanei          (88)  KADLVNNLGTIAKSGTKAFMEALQAGADISMIGQFGVGFYSAFLVADKVVVTSKNNDDESYQWESSAGGSFVVRPYNDPE
C.briggsae         (88)  KADLVNNLGTIAKSGTKAFMEALQAGADISMIGQFGVGFYSAFLVADKVVVTSKNNDDDSYQWESSAGGSFVVRPYNDPE
C.japonica        (107)  --------------------EALQAGADISMIGQFGVGFYSAFLVADKVVVTSKSNDDDSYQWESSAGGSFVVRQYNDPE
G.rostochiensis          --------------------------------------------------------------------------------
G.pallida          (89)  KADLVNNLGTIAKSGTKAFMEALQAGADISMIGQFGVGFYSAFLIADRVTVISKHNDDDCYQWESSAGGSFIIRPCSDPE
H.glycines         (88)  KADLVNNLGTIAKSGTKAFMEALQAGADISMIGQFGVGFYSAFLVADRVTVTSKHNDDDCYQWESSAGGSFIIRNCADPE
M.hapla            (83)  KADLVNNLGTIAKSGTKAFMEALQAGADISMIGQFGVGFYSAFLVADRVIVTSKHNDDDCHQWESSAGGSFIIRNCVDPE
Consensus/80%            KADLVNNLGTIAKSGTKAFMEALQAGADISMIGQFGVGFYSAFLVAD+VlVsSKpNDDDsYQWESSAGGSFllR.hsDPE
```

```
B.pahangi         (170) LTRGTKITLYIKEDQTDYLEERRIKEIVKKHSQFIGYPIKLTVEKERDK EVSDDEAEEEKK-DE---DKEKKEGEIEDVG
T.cati            (173) VTRGTKITLHIKEDQTEYLEERKIKEIVKKHSQFIGYPIKLTVEKEREK EVSDDEAEEEKK-DE---GKEKKEGEIEDVG
L.sigmondontis    (170) LTRGTKITLYIKEDQTDYLEERRIKEIVKKHSQFIGYPIKLTVEKERDK EVSDDEAEEDKK-DE---DKEKKEGEIEDVG
A.suum            (171) VTRGTKITLHIKEDQTEYLEERKIKEIVKKHSQFIGYPIKLTVEKEREK EVSDDEAEEEKK-DE---GKEKKEGEIEDIG
H.contortus       (168) VTRGTKITMHIKEDQTEVLEERRIKEIVKKHSQFIGYPIKLVVEKEREK EVEDDEAEETK-E-------EAKEGEVENVG
P.pacificus       (163) VTRGTKITMHIKEDQIEYLEERKIKEIIKKHSQFIGYPIKLVVEKEREK EVEDDEADEEK-E-------EKKEGEVENVG
C.brenneri        (168) LTRGTKITMYIKEDQVDFLEERKIKEIVKKHSQFIGYPIKLVVEKEREK EVEDEEAVEAKDE-------EKKEGEVENVG
C.elegans         (168) VTRGTKIVMHIKEDQIDFLEERKIKEIVKKHSQFIGYPIKLVVEKEREK EVEDEEAVEAKDE-------EKKEGEVENVA
C.remanei         (168) LTRGTKITMYIKEDQIDFLEERKIKEIVKKHSQFIGYPIKLVVEKEREK EVEDEEAVEAKDE-------EKKEGEVENVE
C.briggsae        (168) LTRGTKITMYIKEDQVDFLEERKIKEIVKKHSQFIGYPIKLVVEKEREK EVEDEEAVESKDE-------EKKEGDVENVG
C.japonica        (168) LTRGTKITMYIKDDQIDFLEERKIKEIVKKHSQFIGYPIKLLVEKEREK EVEDEEAEETKEE-------EEKEGEVENVG
G.rostochiensis         ------------------------------------------------- -------------------------------
G.pallida         (169) VTRGTKITLHLKEDQTDYLEERRVREVVKKHSQFIGYPIKL-------- -------------------------------
H.glycines        (168) VTRGTKIVLHLKEDQTDYLEERRVREVVKKHPQFIGYPIKLLVEKERDK EISDDEAEEEKKEDE---AKEEEKKPEDDVS
M.hapla           (163) MTRGTKITLFLKEDQTDYLEERRIREVVKKHSQFIGYPIKLLVEKERDK EISDDEAEDEKKEDK(7)EKEEKKEDEEKEG
Consensus/80%           lTRGTKITbalKEDQh-aLEER+I+ElVKKHSQFIGYPIKLhVEKER-K ElpD-EA.E.K.........EcKc.-.Eslt

B.pahangi         (246) EDEEE-DKK--DKDK-KKKKI KEKYHEDEELNKTKPIWTRNPDDISNEEYAEFYKSLSNDWEDHLAVKHFSVEGQLEFRA
T.cati            (249) EDEDE-DKKDKDKDKKKKKKI KEKYHEDEELNKTKPIWTRNPDDISNEEYAEFYKSLSNDWEDHLAVKHFSVEGQLEFRA
L.sigmondontis    (246) EDEDE-DKK--DKDKKKKKKI KEKYHEDEELNKTKPIWTRNPDDISNEEYAEFYKSLSNDWEDHLAVKHFSVEGQLEFRA
A.suum            (247) EDEDE-DKKDKDKDKKKKKKI KEKYHEDEELNKTKPIWTRNPDDISNEEYAEFYKSLSNDWEDHLAVKHFSVEGQLEFRA
H.contortus       (240) EDEDA-DK-K----KKKTKKI KEKYHEDEELNKTKPIWTRNPDDISNEEYAEFYKSLSNDWEDHLAVKHFSVEGQLEFRA
P.pacificus       (234) EDEDA-DKKN----EKKTKKI KEKYTEDEELNKQKPIWTRNPDDISNEEYAEFYKSLSN-WEDHLAVKHFSVEGQLEFRA
C.brenneri        (241) EDADA-EK-D----KKKTKKI KEKYFEDEELNKTKPIWTRNPDDISNEEYAEFYKSLSNDWEDHLAVKHFSVEGQLEFRA
C.elegans         (241) DDA------D----KKKTKKI KEKYFEDEELNKTKPIWTRNPDDISNEEYAEFYKSLSNDWEDHLAVKHFSVEGQLEFRA
C.remanei         (241) DDA------E----KKKTKKI KEKYFEDEELNKTKPIWTRNPDDISNEEYAEFYKSLSNDWEDHLAVKHFSVEGQLEFRA
C.briggsae        (241) EDADA-EK-D----KKKTKKI KEKYFEDEELNKTKPIWTRNPDDISNEEYAEFYKSLSNDWEDHLAVKHFSVEGQLEFRA
C.japonica        (241) EDADA-EKTD----KKKTKKI KEKYTDEEELNKTKPIWTRNPDDISNEEYAEFYKSLSNDWEDHLAVKHFSVEGQLEFRA
G.rostochiensis     (3) --------------------- ---------------------------------KSLSNDWEDHLAVKHFSVEGQLEFRA
G.pallida               --------------------- -----------------------------------------------------------
H.glycines        (245) DDEAEKKKEEGDKKKKKTKKI KEKYTEDEELNKTKPIWTRNPDDISNEEYAEFYKSLSNDWEDHLAVKHFSVEGQLEFRA
M.hapla           (247) EDEEDKDKKDGEKKKK-TKKI KEKYTEDEELNKTKPIWTRNPDDITNEEYAEFYKSLSNDWEDHLAVKHLSVEGQLEFRA
Consensus/80%           -D............KKKpKKI KEKYhEDEELNKTKPIWTRNPDDISNEEYAEFYKSLSNDWEDHLAVKHFSVEGQLEFRA
```

```
B.pahangi         (322) LLFVPQRAPFDLFENKKTKNAIKLYVRRVFIMENCDELMPEYLNFIKGVVDSEDLPLNISREMLQQSKILKVIRKNLVKK
T.cati            (328) LLFVPQRAPFDLFENKKTKNAIKLYVRRVFIMENCDELMPEYLNFIKGVVDSEDLPLNISREMLQQSKILKVIRKNLVKK
L.sigmondontis    (323) LLFVPQRAPFDLFENKKTKNAIKLYVRRVFIMENCDELMPEYLNFIKGVVDSEDLPLNISREMLQQSKILKVIRKNLVKK
A.suum            (325) LLFVPQRAPFDLFENKKTKNAIKLYVRRVFIMENCDELMPEYLNFIKGVVDSEDLPLNISREMLQQSKILKVIRKNLVKK
H.contortus       (314) LLFVPQRAPFDLFENKKNKNSIK-YVRRVFIMENCEELMPEYLNFIKGVVDSEDLPLNISREMLQQSKILKVIRKNLVKK
P.pacificus       (308) LLYVPQRAPFDLFENKKAKNSIKLYVRRVFIMENCDELMPEYLNFVRGVVDSEDLPLNISREMLQQSKILKVIRKNLVKK
C.brenneri        (315) LLFAPQRAPFDLFENKKSKNSIKLYVRRVFIMENCEELMPEYLNFIKGVVDSEDLPLNISREMLQQSKILKVIRKNLVKK
C.elegans         (311) LLFVPQRAPFDLFENKKSKNSIKLYVRRVFIMENCEELMPEYLNFIKGVVDSEDLPLNISREMLQQSKILKVIRKNLVKK
C.remanei         (311) LLFAPQRAPFDLFENKKSKNSIKLYVRRVFIMENCEELMPEYLNFIRGVVDSEDLPLNISREMLQQSKILKVIRKNLVKK
C.briggsae        (315) LLFAPQRAPFDLFENKKSKNSIKLYVRRVFIMENCEELMPEYLNFIKGVVDSEDLPLNISREMLQQSKILKVIRKNLVKK
C.japonica        (316) LLFVPQRAPFDLFENKKTKNSIKLYVRRVFIMENCEELMPEYLNFIKGVVDSEDLPLNISREMLQQSKILKVIRKNLVKK
G.rostochiensis    (29) LLFVPQRAPFDLFENKKSKNAIKLYVRRVFIMENCEELMPEYLNFVKGVVDSEDLPLNISRETLQQSKILKVIRKNLVKK
G.pallida         (210) -----------------------------------------------------------------QSKILKVIRKNLVKK
H.glycines        (328) LLFVPQRAPFDLFENKKSKNAIKLYVRRVFIMENCEELMPEYLNFIKGVVDSEDLPLNISRETLQQSKILKVIRKNLVKK
M.hapla           (328) LLFVPQRAPFDMFENKKQKNAIKLYVRRVFIMENCEELMPEYLNFIKGVVDSEDLPLNISREMLQQSKILKVIRKNLVKK
Consensus/80%           LLFsPQRAPFDLFENKKsKNtIKLYVRRVFIMENC-ELMPEYLNFIKGVVDSEDLPLNISREMLQQSKILKVIRKNLVKK

B.pahangi         (402) CLELFDEIAEDKDNFKKFYEQFSKNIKLGIHEDSTNRKKLSEFLRFYTSASSEEMTSLKDYVSRMKENQKQIYFITGESR
T.cati            (408) CLELFDEIAEDKDNFKKFYEQFSKNIKLGIHEDSTNRKKLAEFLRFYTSNSAEELCSLKDYVSRMKENQKQIYFITGETK
L.sigmondontis    (403) CLELFDEIAEDKDNFKKFYEQFSKNIKLGIHEDSTNRKKLSEFLRFYTSASSEEMTSLKDYVSRMKENQKQIYFITGESR
A.suum            (406) CLELFDEIAEDKDNFKKFYEQFSKNIKLGIHEDSTNRKKLAEFLRFYTSNSPEEMCSLKDYVGRMKENQKQIYFITGESK
H.contortus       (393) CLELFEEIAEDKDNFKKFYEQFGKNIKLGIHEDSTNRKKMADFLRYYSSSSPDEQTSLKDYVSRMKDNQTQIYYITGESK
P.pacificus       (388) CIELFEEIAEDKDNFKKFYEHFGKNLKLGIHEDSTNRKKLADFLRYSSSTSGEEVTSLKDYVSRMKENQSQIYYITGESK
C.brenneri        (395) CMELFDEIAEDKDNFKKFYEQFGKNLKLGIHEDSTNRKKLAEFLRY-SSSAGEEPTSLKDYVSRMKENQTQIYYITGESK
C.elegans         (391) CMELIDEVAEDKDNFKKFYEQFGKNLKLGIHEDSTNRKKLSDFLRY-STSAGDEPTSLKEYVSRMKENQTQIYYITGESK
C.remanei         (391) CMELFDEIAEDKDNFKKFYEQFGKNLKLGIHEDSTNRKKLSEFLRY-STSAGEEPTSLKEYVSRMKENQTQIYYITGESK
C.briggsae        (395) CMELFDEIAEDKDNFKKFYEQFGKNLKLGIHEDSTNRKKLSEFLRY-ATSAGEEPTSLKEYVSRMKENQTQIYYITGESK
C.japonica        (396) CMELFDEIAEDKDNFKKFYEQFGKNVKLGIHEDSTNRKKLSEFLRY-STSAGEEPTSLKDYVSRMKENQTQIYYITGESK
G.rostochiensis   (109) CMELFEEIAEDKENFKKFYEQFAKNIKLAIHEDSVNRKKLSEFLRYHTSASGEETCGLKDYVSRMKENQTCIYYITGESK
G.pallida         (225) CMELFEEIAEDKENFKKFYEQFAKNIKLAIHEDSVNRKKLSEFLRYHTSASGEETCGLKDYVSRMKENQTCIYYITGESK
H.glycines        (405) CMDLFEEISEDKDNFKKFYEQFAKNIKLGIHEDSVNRKKLSDFLRYYTSASGEEPCSFKDYVSRMKENQTCIYYITGESK
M.hapla           (406) CVELFDEIAEDKDNFKKFYEQFSKNLKLGIHEDSVNRKKLAEYLRYNTSSSADELVSLKDYVGRMKENQTCIYYITGESK
Consensus/80%           CbELF-EIAEDKDNFKKFYEQFtKNlKLGIHEDSsNRKKLt-FLRa.**tttEE.sSLKDYVSRMKENQppIYaITGESK
```

```
B.pahangi         (482) EAVASSAFVERVKRRGFEVIYMTDPIDEYCVQQLKEYDGKKLVSVTKEGLELPESEEEKKKFEEDKV KFENLCKVMKDIL
T.cati            (488) EAVASSAFVERVKRRGFEVVYMTDPIDEYCVQQLKEYDGKKLVSVTKEGLELPESEEEKKKFEEDKV KYESLCKVIKDIL
L.sigmondontis    (483) EAVASSAFVERVKRRGFEVVYMTDPIDEYCVQQLKEYDGNKLVSVTKEGLELPESEEEKKKFEEDKV KFENLCKVMKDIL
A.suum            (486) ESVASSAFVERVKRRGFGVIYMTDPIDEYCVQQLKEYDGKKLVSVTKEGLELPESEEEKKKFEEDNV KYENLCKVIKDIL
H.contortus       (473) DAVANSAFVERVRNRGFEVLYMVDPIDEYCVQQLKEYEGKKLVSVTKEGLELPESEDEKKKFEEDKV KFENLCKVIKDIL
P.pacificus       (468) DAVANSAFVERVRKGGFEVLYMVDPIDEYCVQQLKEYDGKKLVSVTKEGLELPTSEEQKKKFEEDKV KFENLCKAVKDIL
C.brenneri        (474) EVVAASAFVERVKSRGFEVLYMCDPIDEYCVQQLKEYDGKKLVSVTKEGLELPETEEEKKKFEEDKV AYENLCKVIKDIL
C.elegans         (470) DVVAASAFVERVKSRGFEVLYMCDPIDEYCVQQLKEYDGKKLVSVTKEGLELPETEEEKKKFEEDKV AYENLCKVIKDIL
C.remanei         (470) EVVAASAFVERVKSRGFEVLYMCDPIDEYCVQQLKEYDGKKLVSVTKEGLELPETEEEKKKFEEDKV AYENLCKVIKDIL
C.briggsae        (474) EVVAASAFVERVKSRGFEVLYMCDPIDEYCVQQLKEYDGKKLVSVTKEGLELPETEEEKKKFEEDKV AYENLCKVIKDIL
C.japonica        (475) EVVAASAFVERVKSRGFEVLYMCDPIDEYCVQQLKEYDGKKLVSVTKEGLELPESEDEKKKFEEDKV KYENLCKVIKDIL
G.rostochiensis   (189) DVVQNSSFVERVKKRGFEVIYMVDPIDEYCVQQLKVFDGKKLVSVTKEGLELPESEEEKKKFEEDKV KFEKLCKVVKDIL
G.pallida         (305) DVVQNSSFVERVKKRGFEVIYMVDPIDEYCVQQLKEFDGKKLVSVTKEGLELPESEEEKKKFEEDKV KFEKLCKVVKDIL
H.glycines        (485) DVVQNSSFVERVKKRGFEVIYMVDPIDEYCVQQLKEYDGKKLVSVTKEGLELPESEEEKKKFEEDKV KFEKLCKVIKDIL
M.hapla           (486) EVVQNSAFVERVKKRGFEVIYMVDPIDEYCIQQLKEFDGKKLVSVTKEGLELPESEEEKKKFEEDKV KFEKLCKVIKDIL
Consensus/80%           -sV.sSAFVERVKpRGFEVlYMsDPIDEYCVQQLKEYDGKKLVSVTKEGLELPE*EEEKKKFEEDKV .aEpLCKVlKDIL

B.pahangi         (562) EKKVEKVAVSNRLVSSPCCIVTSEYGWSANMERIMKAQALRDSSTMGYMAAKKHLEINPDHSVIKALRERVEADKNDKTV
T.cati            (562) EKKVEKVAVSNRLVSSPCCIVTSEYGWSANMERIMKAQALRDSSTMGYMAAKKHLEINPDHSVIKALRERVEADKNDKTV
L.sigmondontis    (563) EKKVEKVAVSNRLVSSPCCIVTSEYGWSANMERIMKAQALRDSSTMGYMAAKKHLEINPDHSVIKALRERVEADKNDKTV
A.suum            (566) EKNVEKVVVSNRLVSSPCCIVTSEYGWSANMERIMKAQALRDSSTMGYMAAKKHLEINPDHSVIKALRERVEADKNDKTV
H.contortus       (553) EKKVEKVVVSNRLVHSPCCIVTSEYGWSANMERIMKAQALRDSSTMGYMAAKKHLEINPDHAIM-TLRERVEVDKNDKTV
P.pacificus       (548) EKKVEKVAVSERLVSSPCCIVTSEYGWSANMERIMKAQALRDSSTMGYMAAKKHLEINPDHAII-TLAERVEADKNDKTV
C.brenneri        (564) EKKIEKVAVSNRLVSSPCCIVTSEYGWSANMERIMKAQALRDSSTMGYMAAKKHLEINPDHAIMKTLRERVEADKNDKTV
C.elegans         (560) EKKVEKVGVSNRLVSSPCCIVTSEYGWSANMERIMKAQALRDSSTMGYMAAKKHLEINPDHAIMKTLRDRVEVDKNDKTV
C.remanei         (560) EKKIEKVAVSNRLVSSPCCIVTSEYGWSANMERIMKAQALRDSSTMGYMAAKKHLEINPDHAIMKTLRERVETDKNDKTV
C.briggsae        (564) EKKIEKVAVSNRLVSSPCCIVTSEYGWSANMERIMKAQALRDSSTMGYMAAKKHLEINPDHAIMKTLRERVEADKNDKTV
C.japonica        (555) EKKIEKVAVSNRLVSSPCCIVTSEYGWSANMERIMKAQALRDSATMGYMAAKKHLEINPDHAIMKTLRDRVEADKNDKTV
G.rostochiensis   (268) DKKVQKVSVSNRLVSSPCCIVTGEYGWTANMERIMKAQALRDSSTMGYMASKKNMEINPDHSIIKSLRERVESDQDDKTA
G.pallida         (385) DKKVQKVSVSNRLVSSPCCIVTGEYGWTANMERIMKAQALRDSSTMGYMASKKNMEINPDHSIIKSLRERVESDQDDKTA
H.glycines        (565) DKKVQKVSVSNRLVSSPCCIVTGEYGWSANMERIMRAQALRDSSTMGYMASKKNLEINPDHSIIKSLRDRVEKEQDDKTA
M.hapla           (566) DKKVQKVSISNRLVSSPCCIVTGEYGWTANMERIMKAQALRDSSTMGYMASKKNLEINPDHSIIKSLRERIDSDQDDKTA
Consensus/80%           -KKlpKVtVSNRLVSSPCCIVTtEYGWSANMERIMKAQALRDSSTMGYMAtKKpLEINPDHtlbKsLRERVEsDpsDKTs
```

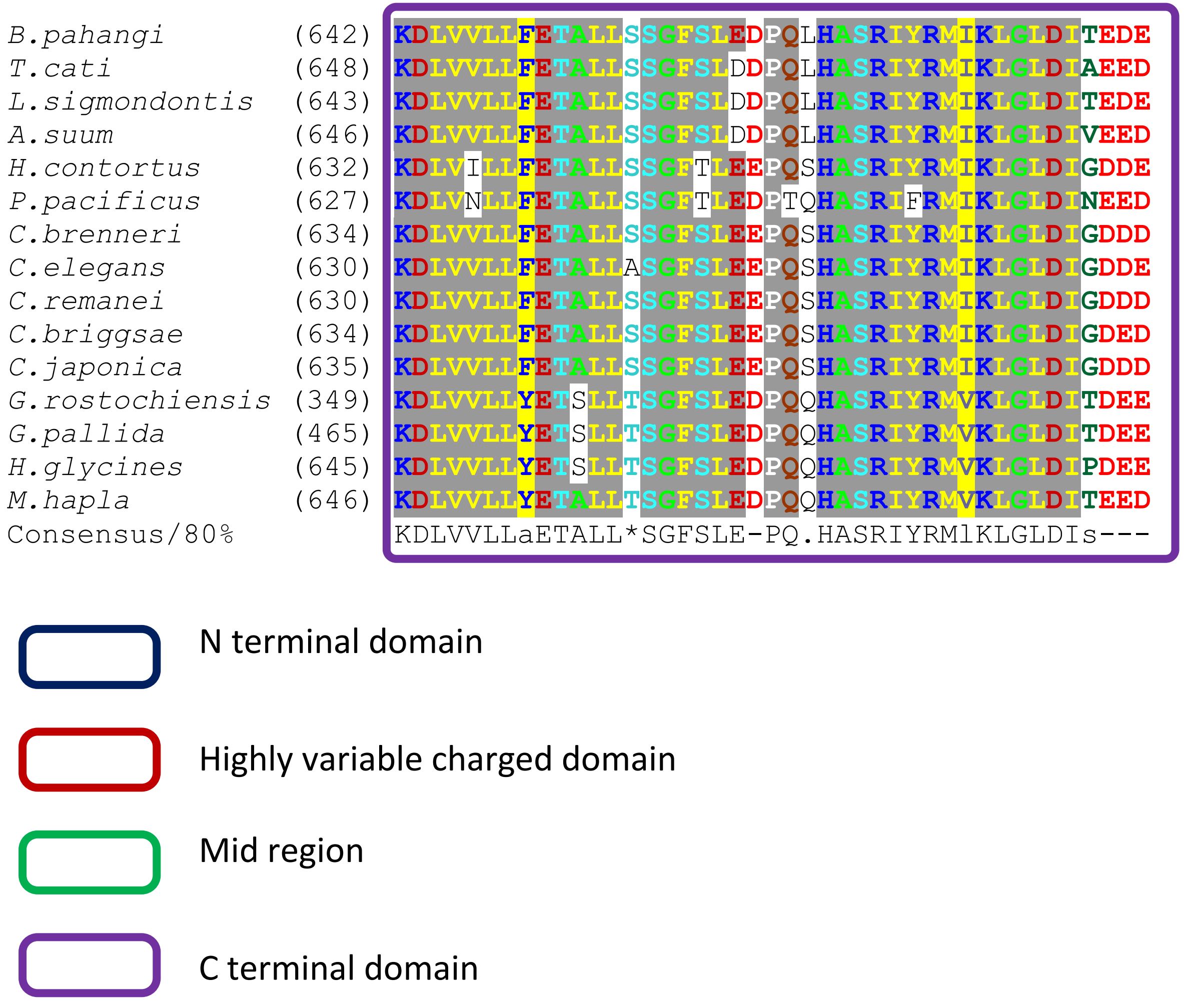

B.pahangi (642) KDLVVLLFETALLSSGFSLEDPQLHASRIYRMIKLGLDITEDE
T.cati (648) KDLVVLLFETALLSSGFSLDDPQLHASRIYRMIKLGLDIAEED
L.sigmondontis (643) KDLVVLLFETALLSSGFSLDDPQLHASRIYRMIKLGLDITEDE
A.suum (646) KDLVVLLFETALLSSGFSLDDPQLHASRIYRMIKLGLDIVEED
H.contortus (632) KDLVILLFETALLSSGFTLEEPQSHASRIYRMIKLGLDIGDDE
P.pacificus (627) KDLVNLLFETALLSSGFTLEDPTQHASRIFRMIKLGLDINEED
C.brenneri (634) KDLVVLLFETALLSSGFSLEEPQSHASRIYRMIKLGLDIGDDD
C.elegans (630) KDLVVLLFETALLASGFSLEEPQSHASRIYRMIKLGLDIGDDE
C.remanei (630) KDLVVLLFETALLSSGFSLEEPQSHASRIYRMIKLGLDIGDDD
C.briggsae (634) KDLVVLLFETALLSSGFSLEEPQSHASRIYRMIKLGLDIGDED
C.japonica (635) KDLVVLLFETALLSSGFSLEEPQSHASRIYRMIKLGLDIGDDD
G.rostochiensis (349) KDLVVLLYETSLLTSGFSLEDPQQHASRIYRMVKLGLDITDEE
G.pallida (465) KDLVVLLYETSLLTSGFSLEDPQQHASRIYRMVKLGLDITDEE
H.glycines (645) KDLVVLLYETSLLTSGFSLEDPQQHASRIYRMVKLGLDIPDEE
M.hapla (646) KDLVVLLYETALLTSGFSLEDPQQHASRIYRMVKLGLDITEED
Consensus/80% KDLVVLLaETALL*SGFSLE-PQ.HASRIYRMlKLGLDIs---
N terminal domain
Highly variable charged domain
Mid region
C terminal domain
