# Supplementary figures and images for: Hsp-90 and the biology of nematodes
Source: BMC Evol Biol. 2009 Oct 22;9:254. doi: 10.1186/1471-2148-9-254 (PMC2771018; doi:10.1186/1471-2148-9-254)

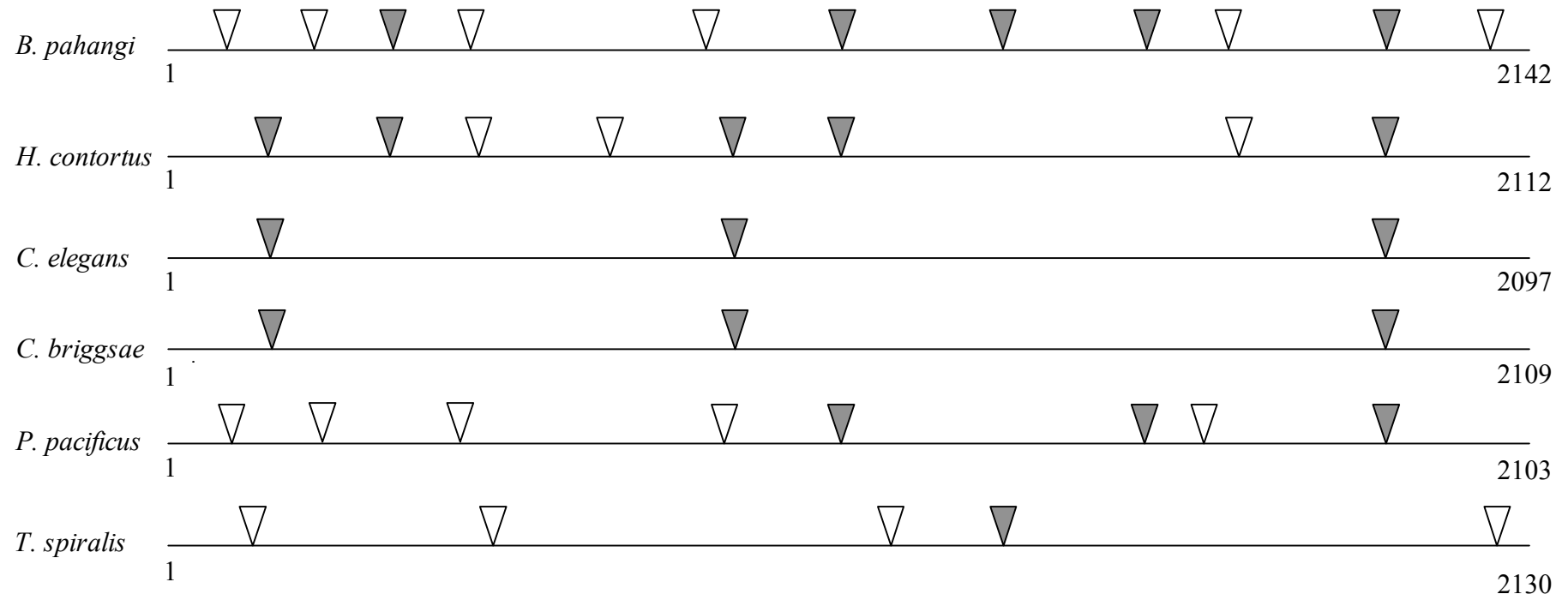

Supplement: Additional file 2 — Genomic structure of nematode hsp-90 genes. The figure shows intron positions in genomic DNA sequences of hsp-90 from B. pahangi, H. contortus, C. elegans, C. briggsae, P. pacificus and T. spiralis. Introns were mapped by comparison of cDNA and genomic DNA sequences. Intron positions were marked by hand and introns with common positions are shaded. Numbers refer to the length of cDNA sequences of each gene. [file 1471-2148-9-254-S2.PDF]
